# Two *LcbHLH* Transcription Factors Interacting with *LcMYB1* in Regulating Late Structural Genes of Anthocyanin Biosynthesis in *Nicotiana* and *Litchi chinensis* During Anthocyanin Accumulation

**DOI:** 10.3389/fpls.2016.00166

**Published:** 2016-02-18

**Authors:** Biao Lai, Li-Na Du, Rui Liu, Bing Hu, Wen-Bing Su, Yong-Hua Qin, Jie-Tang Zhao, Hui-Cong Wang, Gui-Bing Hu

**Affiliations:** ^1^State Key Laboratory for Conservation and Utilization of Subtropical Agro-Bioresources, College of Horticulture, South China Agricultural UniversityGuangzhou, China; ^2^Physiological Laboratory for South China Fruits, College of Horticulture, South China Agricultural UniversityGuangzhou, China

**Keywords:** anthocyanins, MYB, bHLH, interaction, *Litchi chinensis*, tobacco

## Abstract

Anthocyanin biosynthesis requires the MYB-bHLH-WD40 protein complex to activate the late biosynthetic genes. *LcMYB1* was thought to act as key regulator in anthocyanin biosynthesis of litchi. However, basic helix-loop-helix proteins (bHLHs) as partners have not been identified yet. The present study describes the functional characterization of three litchi bHLH candidate anthocyanin regulators, *LcbHLH1*, *LcbHLH2*, and *LcbHLH3*. Although these three litchi bHLHs phylogenetically clustered with bHLH proteins involved in anthcoyanin biosynthesis in other plant, only LcbHLH1 and LcbHLH3 were found to localize in the nucleus and physically interact with *LcMYB1*. The transcription levels of all these bHLHs were not coordinated with anthocyanin accumulation in different tissues and during development. However, when co-infiltrated with *LcMYB1*, both *LcbHLH1* and *LcbHLH3* enhanced anthocyanin accumulation in tobacco leaves with *LcbHLH3* being the best inducer. Significant accumulation of anthocyanins in leaves transformed with the combination of LcMYB1 and LcbHLH3 were noticed, and this was associated with the up-regulation of two tobacco endogenous bHLH regulators, *NtAn1a* and *NtAn1b*, and late structural genes, like *NtDFR* and *NtANS*. Significant activity of the *ANS* promoter was observed in transient expression assays either with *LcMYB1*-*LcbHLH1* or *LcMYB1*-*LcbHLH3*, while only minute activity was detected after transformation with only *LcMYB1*. In contrast, no activity was measured after induction with the combination of *LcbHLH2* and *LcMYB1*. Higher DFR expression was also oberseved in paralleling with higher anthocyanins in co-transformed lines. LcbHLH1 and LcbHLH3 are essential partner of LcMYB1 in regulating the anthocyanin production in tobacco and probably also in litchi. The LcMYB1-LcbHLH complex enhanced anthocyanin accumulation may associate with activating the transcription of *DFR* and *ANS*.

## Introduction

Among the pigments that confer color to plants, anthocyanins are of particular interest because they are not only responsible for most of the red, blue, or black color in plants, but also for the beneficial effects on plant physiological processes and human health (Winkel, 2006). The biosynthetic pathway for anthocyanin biosynthesis has been well characterized and the corresponding genes have been isolated from various plant species (Hichri et al., 2011).

Research on model plants has shown that the expression of structural anthocyanin genes, particularly late genes, are orchestrated by a so-called MBW ternary complex, which is composed of MYB and bHLH transcription factors, together with WD40 repeat proteins (Broun, 2005; Koes et al., 2005; Hichri et al., 2011). In plants, R2R3 MYBs are considered to be key transcription factors known as the regulators of anthocyanin biosynthesis. MYBs in determining anthocyanin biosynthesis have been well characterized in model plants and fruit trees, such as *Arabidopsis* (Borevitz et al., 2000), antirrhinum (Schwinn et al., 2006), petunia (Quattrocchio et al., 1999), apple (Ban et al., 2007; Chagne et al., 2007, 2013), pear (Feng et al., 2010), grape (Kobayashi et al., 2002), litchi (Lai et al., 2014), mangosteen (Palapol et al., 2009), and Chinese bayberry (Niu et al., 2010). The R3 domain of MYBs suggests protein–protein interaction, especially with the bHLH co-factor, also known as MYC (Grotewold et al., 2000; Zimmermann et al., 2004).

The bHLH proteins are also a large class of transcription factors in plants, and have been divided into 26 subgroups (Pires and Dolan, 2010). bHLH transcription factors regulate many cellular processes such as fate of epidermal cells, hormonal response, metal homeostasis, photomorphogenesis, and development of floral organs (Hichri et al., 2011). Flavonoid related bHLHs have been grouped into subgroup IIIf. Maize regulatory gene (*R*) was the first isolated and characterized as a bHLH transcription factor which encodes a protein regulating anthocyanin accumulation (Ludwig et al., 1989, 1990). In *Arabidopsis*, bHLH proteins, TT8, GL3, and EGL3, are involved in production of different flavonoids (Shirley et al., 1995; Payne et al., 2000; Zhang et al., 2003). *NtAn1a* and *NtAn1b* originate from two ancestors of tobacco (*N. sylvestris* and *N. tomentosiformis*) and both enhance anthocyanin accumulation in tobacco flowers (Bai et al., 2011). G to A transition in the bHLH encoding *A* gene is the main reason for white flower color of pea in Mendel genetic research (Hellens et al., 2010). bHLH transcription factors are essential to anthocyanin biosynthesis in plants.

bHLH proteins function as anthocyanin regulator in cultivated fruit species had been reported so far for grape (Hichri et al., 2010), apple (Espley et al., 2007) and Chinese bayberry (Liu et al., 2013). The grape bHLH transcription factors *VvMYC1* and *MYCA1*, were found to be able to induce anthocyanin and proanthocyanidin production through physically interacts with MYBs and consequent activation of the promoters of genes involved in anthocyanin and/or proanthocyanidin synthesis (Hichri et al., 2010). Efficient induction of anthocyanin biosynthesis in transient assays by MdMYB10 was dependent on the co-expression of two distinct bHLH proteins from apple, MdbHLH3 and MdbHLH33 (Espley et al., 2007). Though MrbHLH1 and MrbHLH2 were clustered in IIIf group, only MrbHLH1 was the essential partner of MrMYB1 during anthocyanin biosynthesis regulation in bayberry, the function of MrbHLH2 still unknown (Liu et al., 2013). However, their role in anthocyanin regulation and how they work have not been fully uncovered and the effects of bHLH co-factors in anthocyanin regulation might differ among species (Montefiori et al., 2015; Xu et al., 2015).

The red pigment of litchi pericarp is due to the accumulation of anthocyanins (Lee and Wicker, 1991). *LcMYB1* was thought to act as key regulator in anthocyanin biosynthesis of litchi by activating the late structural genes *UFGT* in particular (Wei et al., 2011; Zhao et al., 2012; Lai et al., 2014). *LcMYB1* can strongly induce anthocyanin biosynthesis in tobacco leaves by its own, without requiring co-infiltration with a *bHLH* partner. However, the upregulation of *NtAn1b* in response to *LcMYB1* overexpression suggested the essential role of bHLH partner in regulating anthocyanin biosynthesis (Lai et al., 2014).

In this study, we isolated three putative litchi bHLH transcription factors, *LcbHLH1*, *LcbHLH2*, and *LcbHLH3*, and analyzed their expression profiles. Phylogenic analysis showed that these three bHLH transcription factors from litchi cluster with bHLH genes related to anthocyanin biosynthesis in other plants. However, expression patterns of these three genes in different litchi tissues and developmental stages do not correlate with anthocyanin contents. BiFC and Y2H assays show that LcbHLH1 and LcbHLH3 can interact *in vivo* with LcMYB1. Transient assays in tobacco leaves showed that both LcbHLH1 and LcbHLH3 enhanced the induction of anthocyanin accumulation by *LcMYB1* with the *LcbHLH3* being by far more efficient. Furthermore, dual LUC assays indicate that the high affinity of LcMYB1 for the promoter of *ANS* induced by LcbHLH3 may associate with enhanced anthocyanin accumulation in tobacco leaves.

## Results

### Identification and Sequence Analysis of Three Candidate Anthocyanin Related bHLH Transcription Factors

**FIGURE 1:**
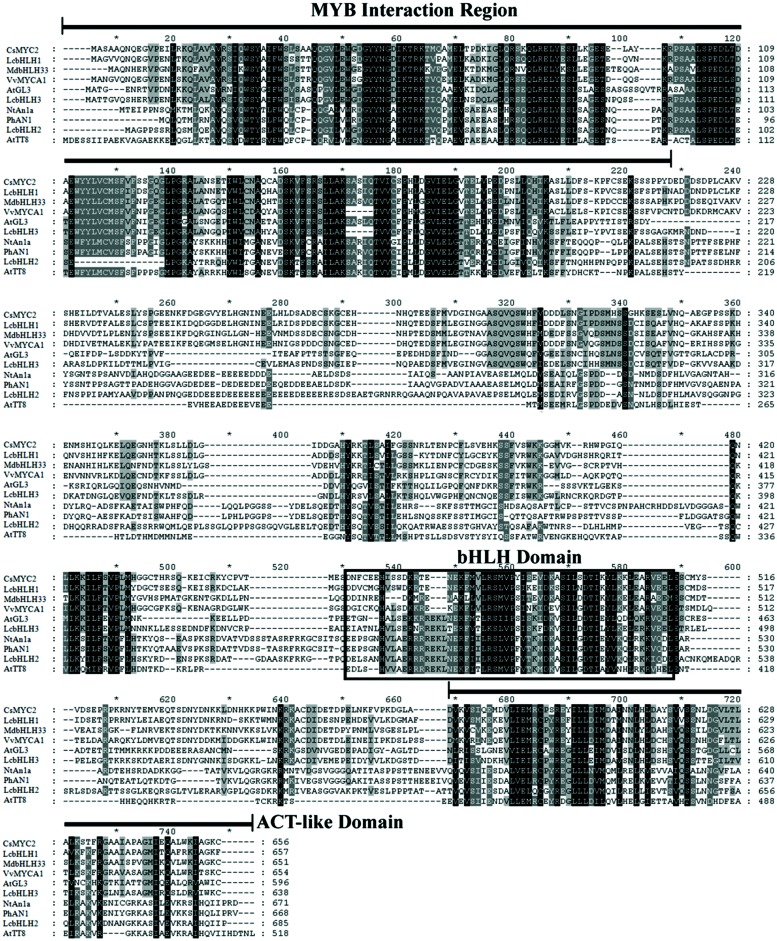
**Protein sequence alignment of three LcbHLH proteins and the known anthocyanin bHLH regulators in other species**. Identical residues are shown in black and conserved residues in dark gray. MYB interaction region, bHLH domain and ACT-like domain are conserved among these bHLH transcription factors.

Three putative members of the bHLH family of transcription factors were identified from the litchi pericarp transcriptomic (Lai et al., 2015) and genomic database^1^, denominated as *LcbHLH1*, *LcbHLH2*, and *LcbHLH3.* The ORFs of *LcbHLH1*, *LcbHLH2*, and *LcbHLH3* encoded proteins with 657, 700, and 643 amino acids, respectively. Three conserved motifs are identified by sequence alignments of LcbHLH1, LcbHLH2, LcbHLH3, and other bHLH transcription factor proteins related to plant anthocyanin biosynthesis (**Figure 1**). The MYB interaction region presented in the N-terminal region of these proteins suggests for all of them protein-protein interaction with MYB transcription factors. A sequence rich in acidic amino acids, containing up to 30 acidic amino acids, is present at the C-terminal region of bHLH proteins (**Figure 1**). This domain was believed to be the transactivation (ACT) domain which interacts with the RNA polymerase II machinery and then initiates transcription (Pattanaik et al., 2008). All three litchi bHLH proteins contained such ACT-like domain, which has also been proven to be involved in the dimerization of plant basic-helix-loop-helix transcription factors (Feller et al., 2006).

**FIGURE 2:**
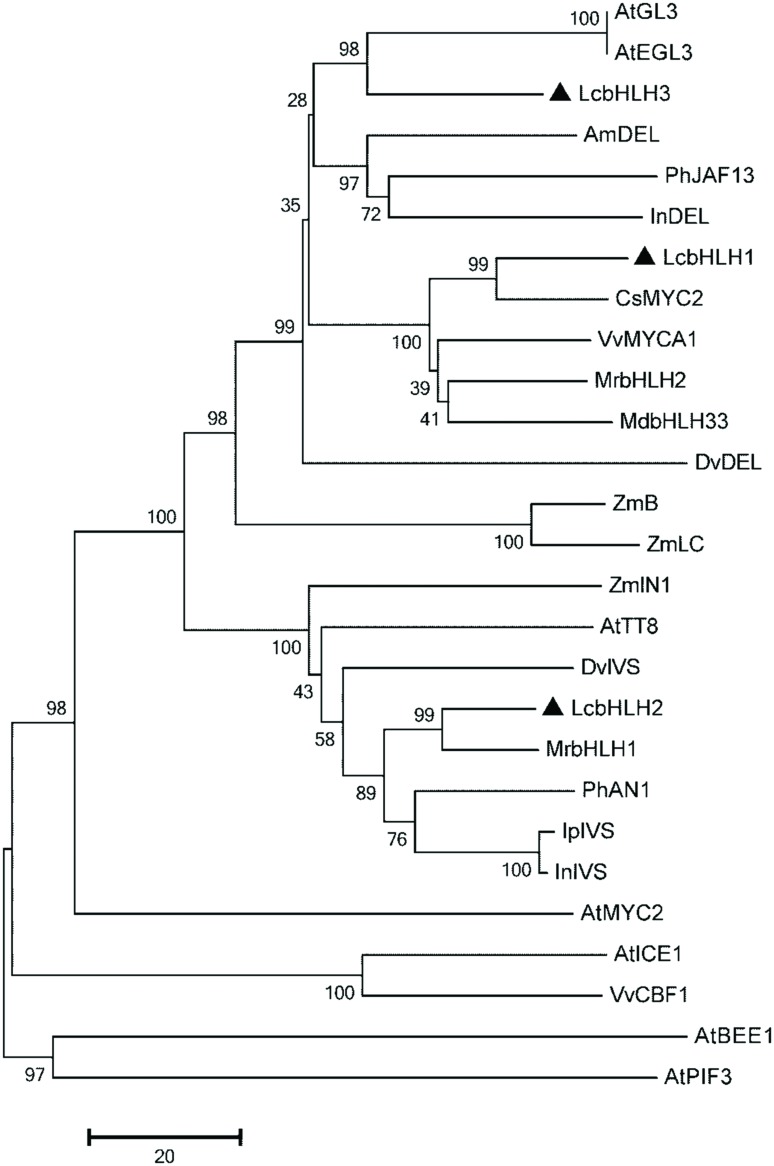
**Phylogenetic relationships between LcbHLH1-3 and anthocyanin-related bHLHs in other species**. The tree was constructed using MEGA 5, neighboring-joining phylogeny testing, and 1,000 bootstrap replicates. The accession number of these proteins (or translated products) are as follows in the GenBank database:AtTT8, CAC14865.1; AtGL3, NP_680372; AtEGL3, NP_176552; MdbHLH33, ABB84474.1; PhJAF13, AAC39455; IpIVS, BAD18982.1; VvMYCA1, NP_001267954.1; CsMYC2, ABR68793.1; PhAN1, AAG25927; AmDEL, AAA32663; DvIVS, BAJ33515; DvDEL, BAJ33516; InDEL, BAE94393; ZmB, AGO65322.1; ZmLC, NP_001105339.1; InIVS, BAE94394; ZmIN1, AAB03841; MrbHLH1, JX629461; MrbHLH2, JX629462; AtMYC2, NP_174541.1; AtICE1, NM_113586.3; VvCBF1, AFI49627.1; AtPIF3, NM_179295.2; AtBEE1, AY138253.1.

A phylogenetic tree constructed with the neighbor-joining method using full-length amino acid sequences showed that the three litchi bHLHs belong to the group IIIf of *Arabidopsis* bHLH which contains bHLH factors involved in anthocyanin and other flavonoid biosynthesis (**Figure 2**) (Heim et al., 2003). The similarity of LcbHLH1 with CsMYC2 (ABR68793.1) and VvMYCA1 (ABM92332) at amino acid level were 67.4 and 60.1%, respectively. LcbHLH2 had 73.1 and 56.2% homology with MrbHLH1 (JX629461) and PhAN1 (AAG25927). LcbHLH3 showed relatively low similarity with AtEGL3 (NP_176552) and VvMYCA1 (NP_001267954.1), 49.9 and 47.7% homology, respectively. The identity between LcbHLH1 and LcbHLH2, LcbHLH1 and LcbHLH3, and LcbHLH2 and LcbHLH3 were 29.6, 47.9 and 28.2%, respectively.

### Subcellular Localization of LcMYB1 and Three Litchi bHLH Proteins

**FIGURE 3:**
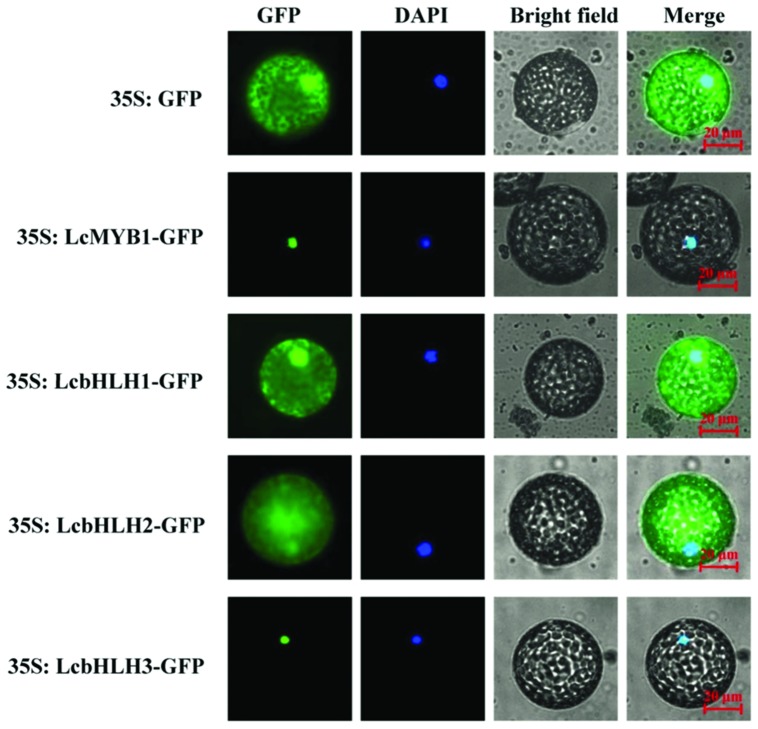
**Subcellular localization of LcMYB1 and LcbHLHs in *N. benthamiana* leaf protoplasts**. Epidermal cells of *N. benthamiana* leaves were transiently transformed with LcMYB1–GFP and LcbHLHs–GFP constructs in *Agrobacterium tumefaciens* strain GV3101. GFP fluorescence was observed with a fluorescence microscope. Images were taken in a dark field for green fluorescence, while the outline of the cell and the merged were photographed in a bright field. Bars, 20 μm.

Basic helix-loop-helix and MYB proteins are TFs and as such are expected to be localized to the nucleus. However, some bHLH proteins are also cytoplasm associated (Hichri et al., 2010). To analyze the subcellular localizations of LcMYB1 and LcbHLH proteins, their full-length coding sequences were fused in frame with the GFP gene. Transient expression of these constructs in epidermal cells of *N. benthamiana* and leaf protoplast showed that the fluorescence for LcMYB1-GFP and LcbHLH3-GFP was localized exclusively in the nucleus. By contrast, fluorescence was observed in the nucleus as well as in the cytoplasm for LcbHLH1-GFP, and LcbHLH2-GFP was mainly localized in the cytoplasm (**Figure 3** and Supplementary Figure S1).

### Interaction of LcMYB1 with Different LcbHLH Partners

**FIGURE 4:**
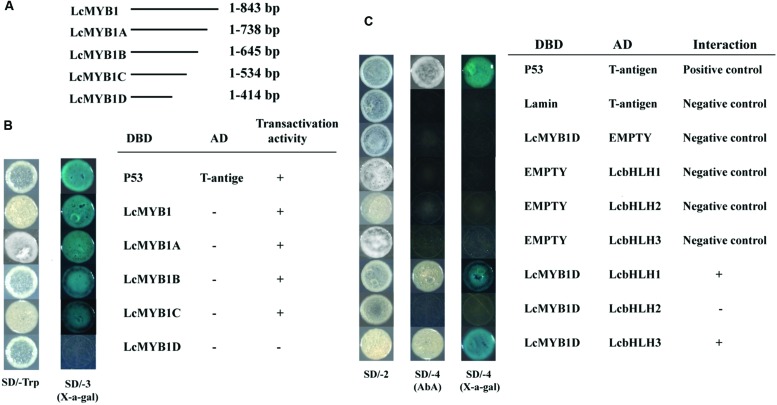
**Physical interactions between LcbHLH1-3 proteins and LcMYB1 detected in Y2H assays**. **(A)** The coding regions of full length and partitial sequence of *LcMYB1* were cloned into the pGBKT7 (GAL4 DBD) vector to create the DBD-LcMYB1 and DBD-LcMYB1A-D constructs, respectively. **(B)** Transcriptional activation analysis of LcMYB1 and partitial sequence of LcMYB1. **(C)** All of the constructs together with the positive control (p-53+T-antigen) and negative control (pGBKT7) were transformed into yeast strain Gold Y2H. Yeast clones transformed with different constructs were grown on SD plates with or without tryptophan, histidine, and adenine but containing 125 μM Aureobasidin A for 3 days at 30°C. Transcription activation was monitored by the detection of yeast growth and a α-galactosidase (α-Gal) assay.

The Y2H was used to investigate the interactions between LcbHLHs and LcMYB1 (**Figure 4**). Expression of the full-length LcMYB1 fused with the DBD resulted in yeast in strong activation (autoactivation) of the reporters. We therefore produced four 3′-deletion fragments and among these, only LcMYB1D (1–402 bp of the LcMYB1 coding sequence) displayed no autoactivation in yeast (**Figures 4A,B**). LcMYB1D was then used in an assay with the three LcbHLHs. As shown in **Figure 4C**, yeast cells co-transformed the positive control (pGBKT7-53+pGADT7-T) and LcbHLH1 or LcbHLH3 with LcMYB1D could grow on selective medium (synthetic medium lacking tryptophan, leucine, histidine, and adenine) supplement with the toxic drug Aureobasidin A, and turned blue in the presence of the chromagenic substrate X-α-Gal. However, yeast cells harboring LcbHLH2 with LcMYB1 and the negative controls, could not grow on the selective medium and did not turn blue under the same conditions. These results suggested that LcMYB1 was able to form complex with either LcbHLH1 or LcbHLH3, but not with LcbHLH2.

**FIGURE 5:**
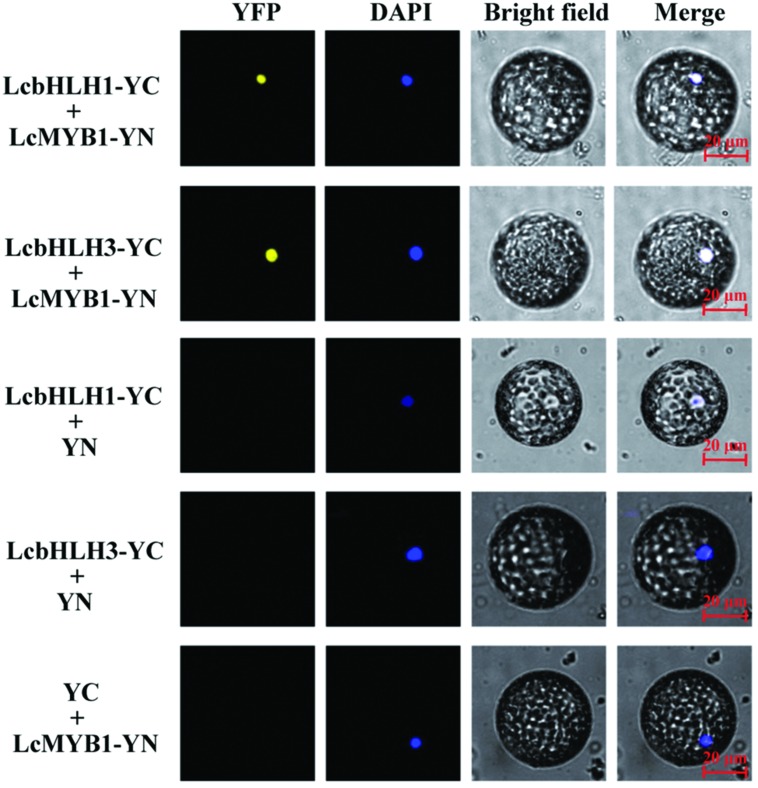
**Bimolecular fluorescence complementation visualization of the LcbHLH1 and LcbHLHs interaction in *N. benthamiana* leaf protoplasts**. YFP indicates fluorescence of YFP; Merge is digital merge of bright field and fluorescent images. Bars, 20 μm.

Subsequently, interaction of LcMYB1 with LcbHLHs was further confirmed in BiFC assay (**Figure 5**). LcMYB1 tagged with split YFP N-terminal fragment (NYFP) and LcbHLH1 or LcbHLH3 tagged with split YFP C-terminal fragment (CYFP) were transiently co-infiltrated in epidermal cells of *N. benthamiana* leaves by *Agrobacterium*. As shown in **Figure 5** and Supplementary Figure S2, strong YFP fluorescent signal was detected in the nucleus of leaf protoplast and epidermal cells expressing LcMYB1-NYFP and LcbHLH1-CYFP fusion protein or LcMYB1-NYFP and LcbHLH3-CYFP, while no YFP fluorescent signal was observed either in the cells expressing the LcMYB1-NYFP with only CYFP, LcbHLH1-CYFP, or LcbHLH3-CYFP with only NYFP. No YFP signal was observed when transformed with both LcMYB1-NYFP and LcbHLH2-CYFP (data not shown). The BiFC assay not only demonstrated the *in vivo* interaction among the three proteins tested but also showed the localization of the interacting proteins, which was consistent with the subcellular localization of LcMYB1, LcbHLH1 and LcbHLH3.

### Expression of Three Litchi *bHLH*s in Relation to Anthocyanin Accumulation

**FIGURE 6:**
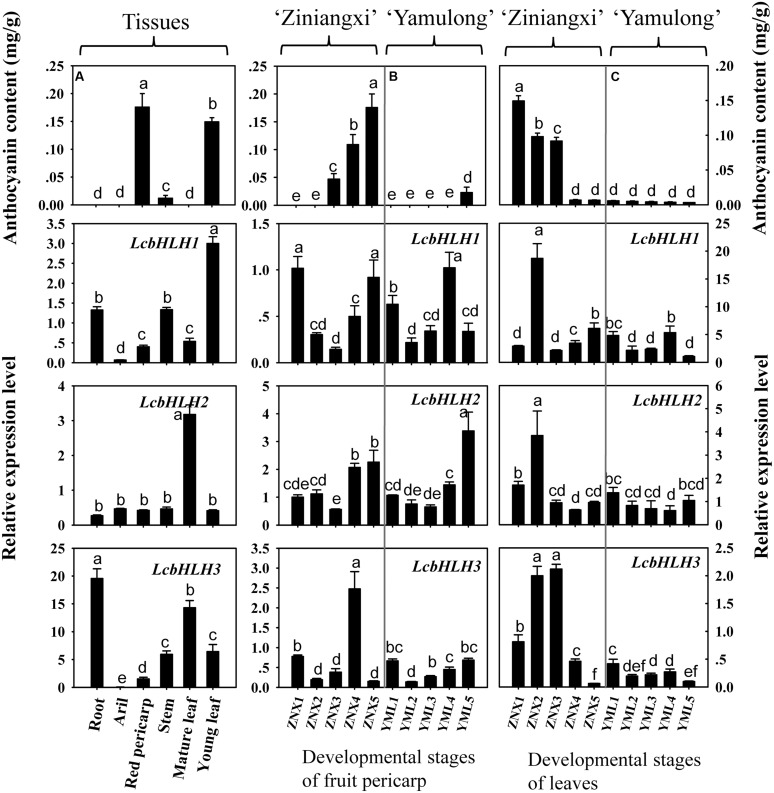
**Expression of three litchi bHLH in relation to anthocyanin accumulation**. **(A)** The transcript patterns of three litchi bHLHs in relation to anthocyanin accumulation among tissues. **(B)** The developmental transcript patterns of three litchi bHLHs in relation to anthocyanin accumulation in the pericarp of ‘Zinianxi’ and ‘Yamulong’. **(C)** The developmental transcript patterns of three litchi bHLHs in relation to anthocyanin accumulation in leaves of ‘Zinianxi’ and ‘Yamulong’. The vertical bars represent the standard error of triplicate experiments. Different letters on the top of columns indicate significant difference at *p* < 0.05.

The transcription of three bHLHs was compared with the anthocyanin accumulation pattern in different tissues (**Figure 6A**). Anthocyanin content varies among different tissues in litchi. No anthocyanin is detectable in root, aril, stems and mature leaf of litchi, while mature pericarp and young leaf accumulated significant amount of anthocyanins. The expression patterns of *LcbHLH1*, *LcbHLH2*, and *LcbHLH3* were not parallel to the accumulation of anthocyanins in any of the analyzed tissues. The transcript amount for all three genes was actually lower in pigmented tissues than non-pigmented tissues.

The developmental patterns of transcript accumulation for the three bHLHs in relation to anthocyanin accumulation were also investigated, specifically in the pericarp of the strongly pigmented cultivar Ziniangxi (ZNX) and of the non-red cultivar Yamulong (YML) (**Figure 6B**). In agreement with the fruit appearance (Supplementary Figure S3), significant accumulation of anthocyanin occurred during fruit maturation in the cultivar ZNX, while only minute anthocyanin amounts were detected in the pericarp of the cultivar YML at maturity. However, comparable levels of *LcbHLH1* and *LcbHLH2* expressions were observed in the pericarp of the two cultivars tested with no apparent trend following pigment accumulation during fruit development. Except for the forth developmental stages of ZNX, the expression of *LcbHLH3* was low in the pericarp of both cultivars.

In the present study, the developmental transcript patterns of three bHLHs in relation to anthocyanin accumulation were also investigated in leaves of the above mention two cultivars (**Figure 6C** and Supplementary Figure S4). The concentrations of anthocyanins were high in young leaves of ZNX, but decreased with leaf development. By contrast, little anthocyanin was detected in the leaves of the non-red cultivar YML throughout leaf development. *LcbHLH1* and *LcbHLH2* were highly expressed in the second developmental stage of the leaves of ZNX, while comparable expression levels were observed during the rest of development and between two cultivars. *LcbHLH3* displayed different transcript accumulation patterns. The expressions of *LcbHLH3* were much higher in pigmented leaves (young leaves of ZNX) than non-red leaves, i.e., mature leaves of ZNX and leaves of YML.

### Transient Expression of Three *bHLHs* in Combination with *LcMYB1*

**FIGURE 7:**
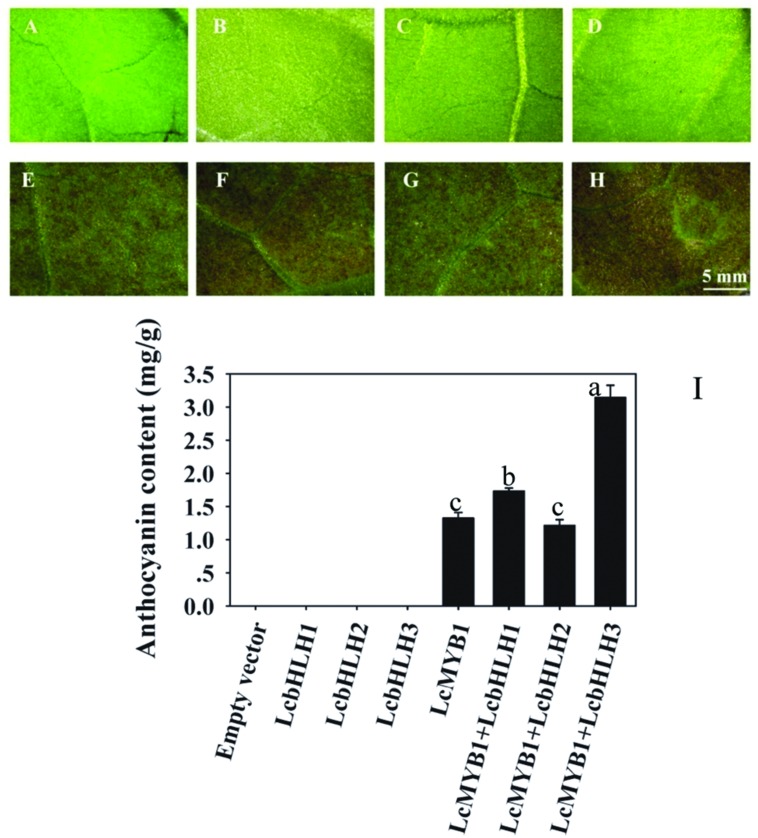
**Anthocyanin accumulation in tobacco leaves infiltrated with *LcMYB1* and co-infiltrated with *LcMYB1* and *LcbHLHs***. **(A)** pEAQ-HT empty vector; **(B)** pEAQ-LcbHLH1; **(C)** pEAQ-LcbHLH2; **(D)** pEAQ-LcbHLH3; **(E)** pEAQ-MYB1; **(F)** pEAQ-MYB1 with pEAQ-LcbHLH1; **(G)** pEAQ-MYB1 with pEAQ-LcbHLH2; **(H)** pEAQ-MYB1 with pEAQ-LcbHLH3. Pictures were taken at 4 days after infiltration. **(I)** Anthocyanin contents in different transiently transformed tobacco leaf patches. The vertical bars represent the standard error of triplicate experiments. Different letters on the top of columns indicate significant difference at *p* < 0.05.

To further characterize the function of three litchi bHLH genes, the ORFs of them were cloned in the transient expression vector pEAQ-HT and transiently transformed into *N. tabacum* leaves via *Agrobacterium* infiltration. Significant anthocyanin accumulation was observed 4 days after infiltration in leaf patches with *LcMYB1* alone as well as co-infiltration with *LcMYB1* or *LcbHLHs*, while no anthocyanin was detected in leaves infiltrated with *LcbHLH1*, *LcbHLH2*, or *LcbHLH3* alone (**Figure 7**). Among the pigmented patches, leaves co-infiltrated with *LcMYB1* and *LcbHLH3* accumulated significantly higher anthocyanin levels than those infiltrated with *LcMYB1* alone and the co-infiltrations of *LcMYB1* and any of the other two bHLH factors.

### The Biosynthesis of Anthocyanins in *LcMYB1* or/and *LcbHLH3* Overexpression Tobacco

**FIGURE 8:**
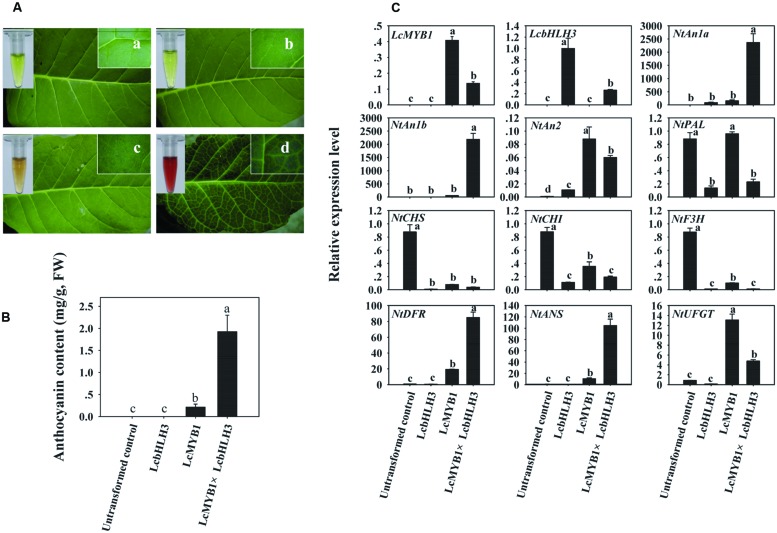
**The biosynthesis of anthocyanins in LcMYB1 or/and LcbHLH3 overexpression tobacco**. **(A)** Color development in leaves of untransformed control and leaves transformation with LcMYB1 or/and LcbHLH3. **(B)** Anthocyanin contents in untransformed control and transgenic tobacco lines. **(C)** The expressions of exogenous litchi regulatory genes and tobacco endogenous regulatory and structural gene in the anthocyanin biosynthetic pathway in transgenic lines. The actin gene was used to normalize gene expression of the genes under identical conditions. The vertical bars represent the standard error of triplicate experiments. Different letters on the top of columns indicate significant difference at *p* < 0.05.

Since *LcHLH3* co-transformed with *LcMYB1* display higher efficiency in inducing anthocyanin accumulation in tobacco leaves as compared to *LcMYB1*-*LcbHLH1*, we produced *LcMYB1*-*LcbHLH3* ectopic expression tobacco lines by crossing a 35S:*LcMYB1* transgenic line with a 35S:*LcbHLH3* line. Transformed tobacco lines ectopically expressing *LcMYB1*, *LcHLH3*, or *LcMYB1*-*LcbHLH3* were grown and used to further investigate the role of these genes in anthocyanin biosynthesis in tobacco. The lines over-expressing *LcMYB1*-*LcbHLH3* accumulated the highest amount of anthocyanin in the leaves, followed by the lines over-expressing *LcMYB1*, while no anthocyanin was detected in untransformed controls and plants expressing *LcbHLH3* (**Figures 8A,B**). Lines expressing the combination of *LcMYB1* and *LcbHLH3* accumulate about 10 times more anthocyanins than lines expressing *LcMYB1* alone.

Furthermore, the expression levels of the transgenes, *LcMYB1* and *LcbHLH3*, and ten anthocyanin biosynthetic genes, including three tobacco anthocyanin regulators, were investigated (**Figure 8C**) in the transgenic lines. Clear *LcMYB1* or/and *LcbHLH3* expression was detected in leaves of the *LcMYB1* or/and *LcbHLH3* transformant lines, while, as expected, no expression was detected in untransformed controls. This result also confirms the successful transformation of *LcMYB1* or/and *LcbHLH3*. The transcript levels for the two tobacco endogenous *bHLH* regulators, *NtAn1a* and *NtAn1b*, were dramatically up-regulated by the combined expression of *LcMYB1* and *LcHLH3*. Early structural genes including *NtPAL*, *NtCHS*, *NtCHI*, and *NtF3H* were down-regulated in *LcMYB1* or/and *LcbHLH3* overexpression leaves, while *NtDFR* and *NtANS* were up-regulated dramatically in leaves of *LcMYB1*-*LcbHLH3* transgenics as compared with lines transformed with *LcMYB1* only. The transcript levels of *NtDFR* and *NtANS* in leaves of *LcMYB1*-*LcbHLH3* transgenics were, respectively, about four and ten times higher than in *LcMYB1* transgenic leaves.

### LcMYB1 and LcMYB1-LcbHLHs Activate the Promoters of Structural Genes

**FIGURE 9:**
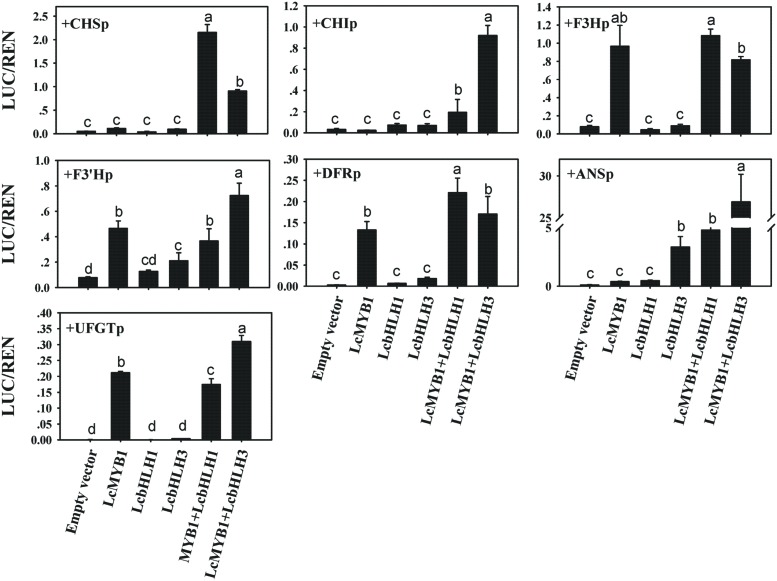
***In vivo* interactions between litchi transcriptional factors and promoters of anthocyanin biosynthetic genes in litchi studied by dual luciferase assay in *N. benthamiana* leaves**. *In vivo* associations of MYB, bHLHs and anthocyanin biosynthetic gene promoters as revealed by transient assays. The vertical bars represent the standard error of four replicate reactions. Different letters on the top of columns indicate significant difference at *p* < 0.05.

Transcription factors modulate the biosynthesis of flavonoids mainly activating the promoters of structural anthocyanin genes (Nesi et al., 2000; Bai et al., 2011; Liu et al., 2013). E-BOX and MYB-CORE *cis*-elements were believed to be the target of bHLH and MYB transcription factors (Hichri et al., 2011; Xu et al., 2015). Lots of E-BOX and MYB-CORE *cis*-elements in the promoter of litchi anthocyanin biosynthesis genes were found (Supplementary Figure S4). In the present study, a dual LUC assay was employed to investigate the downstream target gene of LcMYB1 and LcbHLHs. LcMYB1 or/and LcbHLHs were cloned into pEAQ-HT transient expressing vector as effectors and promoters of structural genes driving LUC gene served as reporters. Different combinations of effector and reporter were transiently expressed in tobacco leaves by *Agrobacterium* based infiltration. As shown in **Figure 9**, four (*LcF3H*, *LcF3′H*, *LcDFR*, and *LcUFGT* promoters) out of the seven investigated promoters were activated by LcMYB1. LcbHLH1 alone did not activate any promoters, while LcbHLH3 clearly activated *LcANS* promoters. When *LcHLH1* and *LcHLH3* were co-transformed with *LcMYB1*, the activities of *LcCHS*, *LcCHI* and *LcANS* promoter were much higher as compared with transformed *LcMYB1* only. The activity of the *LcANS* promoter in *LcHLH3* co-transformed with *LcMYB1* was about six or fifty times higher, respectively, compared with *LcHLH1* co-transformed with *LcMYB1* and *LcMYB1* only.

## Discussion

### Characteristics of Litchi bHLH Transcription Factors

MYBs and bHLHs that regulate the anthocyanin biosynthetic pathway have been extensively described in many plant species (Hichri et al., 2011; Jaakola, 2013; Xu et al., 2015). In litchi, LcMYB1 has been identified as the key regulator of anthocyanin biosynthesis (Lai et al., 2014). However, bHLH interaction partners of this MYB factor have so far not been described in litchi. In the present study, three putative LcbHLH transcription factors were isolated from litchi pericarp (**Figure 1**). The three transcription factors were quite different from each other, with the highest sequence similarity 47.9% between LcbHLH1 and LcbHLH3 at the amino acid level. Based on domain comparison and sequence similarity, these three putative LcbHLHs belong to the IIIf subgroup, which related to regulation of anthocyanin and proanthocyanidin biosynthesis and trichome development in plants (Heim et al., 2003). The three isolated putative LcbHLHs showed high similarity in the conserved motifs of bHLHs regulating pigmentation in other plant species (**Figure 2**).

More than one bHLH factor is known in most plants to regulate anthocyanin or proanthocyanidin biosynthesis, i.e., TT8, GL3, and EGL3 in *Arabidopsis* (Nesi et al., 2000; Payne et al., 2000; Zhang et al., 2003), JAF13 and PhAN1 in petunia (Quattrocchio et al., 1998; Spelt et al., 2002), NtAn1a and NtAn1b in tobacco (Bai et al., 2011), VvMYC1 and VvMYCA1 in grape (Hichri et al., 2010; Matus et al., 2010), MdbHLH3 and MdbHLH33 in apple (Espley et al., 2007). There is increasing evidence of specialization of function for the different bHLH proteins within a single species. In *Arabidopsis*, TT8 is involved in regulation of proanthocyanidin biosynthesis, while GL3 and EGL3 are required for seed coat mucilage production, trichomes and root hair spacing (Shirley et al., 1995; Payne et al., 2000; Zhang et al., 2003). In petunia, *JAF13* gene is homologous to *DELILA* (*DEL*) from snapdragon and *R* from maize which has been shown to regulate anthocyanin accumulation (Quattrocchio et al., 1998). *AN1*, another bHLH factor from petunia does not only control pigment synthesis but also vacuolar pH and seed coat development (Quattrocchio et al., 1993; Spelt et al., 2000).

Although all the three identified LcbHLHs contained MYB interaction region, only LcbHLH1 and LcbHLH3 localized in nucleus and displayed physical interaction with LcMYB1 (**Figures 4–6**). LcbHLH2 clustered with MrbHLH1, a key bHLH transcription factor regulating anthocyanin biosynthesis through interaction with MrMYB1 in bayberry (Liu et al., 2013). But, both yeast two-hybrid and BiFC assays showed no interaction between LcbHLH2 and LcMYB1 (**Figures 4** and **5**). These results suggest that LcbHLH1 and LcbHLH3 maybe, interaction partners of LcMYB1 and could play a role in regulating litchi anthocyanin biosynthesis, while LcbHLH2 is possibly not involved in this pathway.

### The LcbHLH Interaction with LcMYB1 Regulated Anthocyanin Synthesis

The expression analysis of the three litchi bHLHs showed that none of them correlates with anthocyanin accumulation in different tissues and different developmental stages (**Figure 6**). This is consistent with what previously observed for *MdbHLH33*, *MdbHLH*3, and *VvMYC1*, which do not follow neither the accumulation of anthocyanins nor the expression pattern of the MYB factor (Espley et al., 2007; Hichri et al., 2010). Furthermore, transient expression of the three LcbHLHs did not induce anthocyanin accumulation in tobacco leaves (**Figure 7**) when not combined with a MYB factor. These results suggested that litchi bHLHs do not directly regulate the biosynthesis of anthocyanins and do not determine the pigment accumulation pattern.

In apple, Chinese bayberry, grape and peach, without the conjunct expression of bHLH partners, MYB genes do not induce anthocyanin when transiently expressed in tobacco leaves or grape cells (Espley et al., 2007; Hichri et al., 2010; Liu et al., 2013; Rahim et al., 2014). However, overexpression of *LcMYB1* alone efficiently induce anthocyanin accumulation in tobacco leaves. The exogenous *LcMYB1* induces indeed the expression of the tobacco endogenous *bHLH* transcription factor, *NtAn1b* (Lai et al., 2014). The study of the way of action of the kiwifruit AcMYB110 revealed different specificity to promote red pigmentation of tobacco leaves depending on the availability of endogenous bHLHs (Montefiori et al., 2015). These facts suggest the essential role of bHLH in anthocyanin biosynthesis.

In this study, we have tested the role of litchi bHLHs in regulating anthocyanin biosynthesis by co-infiltration with *LcMYB1*. Leaves transiently expressing *LcMYB1*-*LcbHLH1* or *LcMYB1*-*LcbHLH3* accumulated significant higher anthocyanins than leaves expressing just *LcMYB1* or *LcMYB1*-*LcbHLH2* (**Figure 7**). This provides further evidence for the involvement of LcbHLH1 and LcbHLH3 in litchi anthocyanin biosynthesis through interaction with LcMYB1. Similarly, transgenic tobacco (*Nicotiana tabacum*) overexpressing a combination of either potato StAN1 (MYB) with StJAF13 (bHLH) or StAN1 with StbHLH1 showed deeper purple pigmentation with respect to AN1 alone (D’Amelia et al., 2014).

### The LcMYB1-LcbHLH Complex Enhanced Anthocyanin Accumulation by Activating Transcription of *ANS* and *DFR*

Significant accumulation of anthocyanin in the lines of *LcMYB1*-*LcbHLH3* was accompanied by dramatically up-regulation of two tobacco endogenous *bHLH* regulators, *NtAn1a* and *NtAn1b* (**Figure 8C**). Bai et al. (2011) indicated that NtAn1 and NtAn2 complex activates the promoters of two key structural genes of the anthocyanin pathway, *DFR* and *CHS*. In the present study, the accumulation of anthocyanin in an *LcMYB1*-*LcbHLH3* tobacco ectopic-expression line is associated with the upreguation of endogenous *bHLHs*. *NtAn1a* and *NtAn1b*. In tobacco, exogenous MYB requires NtAn1 to activate NtJAF13 then to regulate anthocyanin biosynthesis (Montefiori et al., 2015). These results suggest that the regulation of anthocyanin in tobacco might involve multiple bHLH in a hierarchic fashion.

In petunia, the transport of the bHLH protein AN1 factor to the nucleus is necessary for the activation of the transcription of the DFR gene and this is directly induced by the AN1 protein, as shown by the fact that it takes place in the presence of translation inhibitors (Spelt et al., 2000). The expression of the Dahlia *DvF3H*, *DvDFR*, and *DvANS* are repressed by the insertion of a transposon in the *bHLH* gene *DvIVS* (Ohno et al., 2011). In apple, MdbHLH3 binds to the promoters of anthocyanin biosynthesis genes *MdDFR* and *MdUFGT* and the regulatory gene *MdMYB1* to activate their expression (Xie et al., 2012). MrMYB1–MrbHLH1 complex activated *MrCHI*, *MrF3’H*, *MrDFR1*, *MrANS*, and *MrUFGT* promoters of Chinese bayberry (Liu et al., 2013). In the present study, however, the expressions of *NtCHS*, *NtCHI*, and *NtF3H* were almost diminished in the pigmented transformed control leaves (**Figure 8C**). These early structural genes leads to the formation of the dihydro-flavonols, but not necessarily related to the anthocyanin accumulation. This result consistent with previous reports that late structural genes but not early structural genes determined the anthocyanin accumulation (Niu et al., 2010; Liu et al., 2013; Lai et al., 2014). *LcMYB1* control the biosynthesis of anthocyanins in tobacco leaves by activating the expression of *NtDFR*, *NtANS*, and *NtUFGT* (Lai et al., 2014). In the present study, remarkable up regulation of these three late structural genes were notice in LcMYB1 transformed line, but only *NtDFR* and *NtANS* were upregulated in paralleling with higher anthocyanins in *LcMYB1*-*LcbHLH3* overexpression line as compared with *LcMYB1* overexpression line (**Figure 8C**). All this wealth of data suggests that the target genes of LcMYB1 or/and LcbHLH1 and LcbHLH3 are in litchi the homologous genes of the anthocyanin pathway.

To test this possibility, we isolated the promoters of anthocyanin biosynthesis structural genes in litchi and tested their activation by different combinations of factors. LcMYB1 activates the promoters of *LcF3H*, *LcF3′H*, *LcDFR*, and *LcUFGT*, while LcbHLH3 clearly activats the *LcANS* promoter (**Figure 9**). The activity of *LcDFR* and *LcANS* promoter was higher when they were cotransformed with the combination of regulators *LcMYB1*-*LcbHLH1* or *LcMYB1*-*LcbHLH3*, as compared to *LcMYB1* only. This result was consistent with the upregulation of *NtDFR* and *NtANS* in leaves of *LcMYB1*-*LcbHLH3* ectopic expression lines (**Figure 8**). In *Arabidopsis*, the TT8 protein is required for the expression of two flavonoid late biosynthetic genes, *DF*R and *BAN* (Nesi et al., 2000). No expression of *IpDFR* and *IpANS* was detected in seed coats of *ivs* mutants in *Ipomoea purpurea*, indicating they could be the target of the bHLH protein IVS (Park et al., 2007). We measured activity of the *ANS* promoter as induced by *LcMYB1*-*LcbHLH1* and *LcMYB1*-*LcbHLH3* in transient assay, while we could only detect minute activity of the same promoter upon expression of *LcMYB1*. *LcbHLHs* seems therefore to be required for the high expression of *LcANS*. In conclusion, these results indicated that LcMYB1-LcbHLH complex induces anthocyanin biosynthesis by activating transcription of *ANS* and *DFR*, late structural genes in anthocyanin biosynthesis pathway.

## Materials and Methods

### Plant Materials

Five developmental stage fruits of red litchi cultivars ‘Ziniangxi’ (ZNX) and one non-red cultivar ‘Yamulong’ (YML) were used in this study. These trees were grown in the experimental orchard of Hainan academy of agricultural sciences (Haikou, China) received standard horticultural practices, and disease and insect control. Root, young stem, aril, young leaf, and mature leaf were collected from cultivar ‘ZNX’. Pericarp disks of ‘YML’ were collected between May 28th, 2013 and June 17th, 2013 at 5 days intervals. Pericarp disks of ‘ZNX’ were collected between May 8th, 2013 and May 28th, 2013 at 5 days intervals. Different developmental leaves were sampled at 7 days interval from leaf flushing to mature as reflecting by net photosynthetic rate. All samples were immediately frozen in liquid nitrogen and stored at -80°C until use.

Tobacco (*N. tabacum*) was used for transient expression, *N. benthamiana* plants were used for subcellular localization and BiFC assays. Tobacco plants were grown in green houses at 28°C using natural light. *N. benthamiana* plants were grown in green houses at 25°C.

### Anthocyanin Analysis

The total anthocyanin content was determined according to the method developed by Wei et al. (2011), which involves measuring the absorbance (520 nm) of extracts that have been diluted with pH 1.0 and 4.5 buffers.

### RNA Extraction and cDNA Synthesis

Total RNA was extracted from different tissues of litchi and tobacco using the RNA_OUT_ kit (Tiandz, Beijing, China). Contaminating DNA was removed from RNA preparations using TURBO DNA-free^TM^ (Ambion, USA). cDNA was synthesized from total RNA (2 μg) using oligo (dT) primers according to the manufacturer’s instructions of M-MLV (Invitrogen, USA) in 20 μL of total volume.

### Gene Cloning and Sequence Analysis

The cDNAs were synthesized from the total RNA of the mature pericarp of cultivar ‘ZNX’ and used as the PCR templates. PCR-amplified products of appropriate length were cloned into T/A cloning vector pMD^®^20-T (TaKaRa, Japan) and then transformed into *Escherichia coli* DH5α Max Efficiency^®^ Chemically Competent Cells (TaKaRa, Japan). Primers are listed in Supplementary Table S1. Plasmid DNA was isolated from positive *E. coli* cells and then sent to Beijing Genomics Institute for sequencing. Multiple sequence alignment was performed using ClustalX 1.83^2^ and MEGA5 (Tamura et al., 2011).

### Real-Time Quantitative PCR

Total RNA was extracted from the pericarp of litchi and tobacco leaves and first strand cDNA was synthesized as described above. The transcription levels of both the litchi and tobacco anthocyanin biosynthetic genes were analyzed using quantitative real-time PCR (qRT-PCR) as described previously (Lai et al., 2014). The specific qRT-PCR primers were designed using a BatchPrimer3 program listed in Supplementary Table S2 (You et al., 2008). Using these gene-specific primers, each assay amplified a single product of the correct size and demonstrated an acceptable PCR efficiency (approximately 90%). qRT-PCR reactions were normalized to the Ct values for *LcACTIN* (HQ615689) and *LcGAPDH* (JF759907) in litchi (Zhong et al., 2011), and *NtACTIN* (GQ281246) for tobacco. The relative expression levels of the target genes were calculated using the formula 2^–ΔΔCT^ (Livak and Schmittgen, 2001). All biological replicates were measured in triplicate.

### Transient Assays and Stable Transformation of Tobacco

The plasmids used in the transient expression assay were constructed by ligating full-length *LcbHLH1-3* to pEAQ-HT using *Nru* I and *Xho* I. The primers used to amplify the encoding region were listed in Supplementary Table S3. The product was recombined with the linearized vector pEAQ-HT (In-Fusion^TM^ Advantage PCR Cloning Kits; Clontech). pEAQ-MYB1 was constructed previously (Lai et al., 2014). The constructs (pEAQ-LcbHLH1-3) were maintained in *Agrobacterium tumefaciens* strain GV3101. *Agrobacterium* cultures containing the different constructs were infiltrated into the abaxial leaf surface of *N. tabacum*, as described in Sainsbury et al. (2009). Control was infiltrated with empty vector (pEAQ-HT) at the same time. Digital photographs were taken 5 days after infiltration. Full-length of LcbHLH3 was amplified and then ligated with pBI121 vector. The resulting construct (pBI121- LcbHLH3) was introduced into *A. tumefaciens* strain EHA105. The recombinant strains were used to transform *N. tabacum* K326 using the leaf disk method (Horsch et al., 1985).

### Subcellular Localization Analysis

The coding sequences of *LcMYB1* and *LcbHLH1-3* without the stop codon were amplified by PCR (primers are listed in Supplementary Table S4), and recombined into the pEAQ-HT-GFP vector using *Age* I in frame with the GFP sequence (Sainsbury et al., 2009). The fusion constructs and the control GFP vector were transformed into *Agrobacterium* strain GV3101 by freeze-thaw method. *Agrobacterium* cultures containing the 35S: LcMYB1-GFP, 35S: LcbHLH1-GFP, 35S: bHLH2-GFP, and 35S: LcbHLH3-GFP constructs were infiltrated into *N. benthamiana* leaves. Two days after infiltration, leaf protoplasts were isolated according to Schweiger and Schwenkert (2014). The protoplasts were incubated with 0.1 μg ml^–1^ DAPI for 10 min. GFP and DAPI fluorescence were observed with a fluorescence microscope (Zeiss Axio Observer D1). All transient expression assays were repeated at least three times.

### Yeast Two-Hybrid Assay

Yeast two-hybrid assays were performed using the Matchmaker^TM^ Gold Yeast Two-Hybrid System (Clontech). The coding regions of *LcbHLH1*-*3* and *LcMYB1* with different 3′-deletion were cloned into pGADT7 and pGBKT7 to fuse with the AD and DBD, respectively, to create different baits and preys (primers are shown in Supplementary Table S5). Full length of LcMYB1 showed autoactivation in yeast cells. Partial clones of *LcMYB1* (*LcMYB1D*) on the contrary did not show any transcriptional activation activity in yeast cells. Different pairs of bait and prey constructs were co-transformed into yeast strain Gold Y2H using the lithium acetate method, and yeast cells were grown on a (SD/–Leu/–Trp) according to the manufacturer’s protocol (Clontech) for 3 days. Transformed colonies were then plated onto minimal medium quadruple dropout (SD medium with –Leu/–Trp/–His/–Ade) containing 125 μM Aureobasidin A and 4 mg ml^–1^ X-α-Gal at 30°C to test for possible interactions between LcbHLH1-LcbHLH3 and LcMYB1 according to their growth status and the activity of α-galactosidase.

### BIFC Assays

NYFP (175-end) and CYFP (175-end) were amplified from pSAT5(A)-DEST-cEYFP and pSAT5(A)-DEST-cEYFP-N1 which were purchased from TAIR. pEAQ-NYFP-F CAAATTCGCGACCGGTATGGTGAGCAAGGGCGAGG and pEAQ-NYFP-R: AGTTAAAGGCCTCGAGTCAGTCCTCGATGTTGT GG were used for amplify NYFP, pEAQ-CYFP-F: CAAATTCGCGACCGGTGGCAGCGTGCAGCTCGCCGAC and pEAQ-CYFP-R: AGTTAAAGGCCTCGAGTCACTTGTACAGCTCGTCC were used for amplify CYFP. The products were recombined with the vector pEAQ-HT linearized using *Age* I and *Xho* I, and the obtained fragments were named pEAQ-NYFP and pEAQ-CYFP. The coding sequences of *LcMYB1* and *LcbHLH1-3* were amplified without stop codon by PCR (primers are listed in Supplementary Table S6) and then subcloned into pEAQ-NYFP and pEAQ-CYFP using *Age* I (In-Fusion^TM^ Advantage PCR Cloning Kits; Clontech), respectively. All constructed vectors were then transformed in *Agrobacterium* (strain GV3101). Two days after infiltration, leaf protoplasts were isolated as described above. YFP fluorescence was observed 2 days after infiltration with a fluorescence microscope. Expression of target genes alone was used as negative controls. All transient expression assays were repeated at least three times.

### Dual Luciferase Assay of Transiently Transformed Tobacco Leaves

Specific primers were designed based on litchi whole genome sequence to amplify the promoters of anthocyanin biosynthetic genes *LcCHS*, *LcCHI*, *LcF3H*, *LcF3’H*, *LcANS*, *LcDFR*, and *LcUFGT* (primers are shown in Supplementary Table S7). Conserved *cis*-element motifs located in promoters were searched by online software New PLACE^3^ (Higo et al., 1999). The promoters of anthocyanin biosynthetic genes were inserted into the pGreenII 0800-LUC vector at the 5′ end of a LUC gene (Hellens et al., 2005). All constructs were transformed into *Agrobacterium tumefaciens* GV3101. Activation of promoters by TF was measured as ratio of the enzyme activity of firefly LUC, driven by the promoter under investigation, and the REN, driven by CaMV:35S. Six to eight leaves old *N. benthamiana* plants were used for infiltration. Infiltrations, transient expression analysis, and enzyme activity determination of LUC and REN were conducted as described by Hellens et al. (2005).

## Author Contributions

BL performed most of the experiments and data analysis, and wrote the draft of the paper. L-ND, BH, RL, and W-BS carried out part of material collection, RNA extraction and data analysis. Y-HQ and J-TZ participated in the preparation of the manuscript. H-CW and G-BH conceived, designed and coordinated the studies. All authors have read and approved the final manuscript.

## Conflict of Interest Statement

The authors declare that the research was conducted in the absence of any commercial or financial relationships that could be construed as a potential conflict of interest.

## Footnotes

1 http://litchidb.genomics.cn/page/species/index.jsp

2 http://www.ebi.ac.uk

3 https://sogo.dna.affrc.go.jp/cgi-bin/sogo.cgi

## ABBREVIATIONS

AD: activation domain
ANS: anthocyanidin synthase
bHLH: basic helix-loop-helix
BiFC: bimolecular fluorescence complementation
CHI: chalcone isomerase
CHS: chalcone synthase
DAPI: 4′,6-diamidino-2-phenylindole
DBD: DNA-binding domain
DFR: dihydroflavonol 4-reductase
F3H: flavanone 3-hydroxylase
GFP: green fluorescent protein
LUC: luciferase
MBW: MYB-bHLH-WD40 protein complex
PAL: phenylalanine ammonia lyase
REN: renilla luciferase
UFGT: UDP-glucose:flavonoid 3-*O*-glucosyltransferase
Y2H: yeast two-hybrid assay
YFP: yellow fluorescent protein.
