# Supplementary material for: Two LcbHLH Transcription Factors Interacting with LcMYB1 in Regulating Late Structural Genes of Anthocyanin Biosynthesis in Nicotiana and Litchi chinensis During Anthocyanin Accumulation
Source: Front Plant Sci. 2016 Feb 18;7:166. doi: 10.3389/fpls.2016.00166 (PMC4757707; doi:10.3389/fpls.2016.00166)
Supplement: Supplementary file 1 [file Data_Sheet_1.DOCX]

**Supplementary Table S1｜**Primers used for isolation three *bHLH* genes.

| Name | Sequences(5＇-3＇) | Product size (bp) |
| --- | --- | --- |
| bHLH1_F | ATGGCTGGTGTTGTTCAAAATCAGG | 1974 |
| bHLH1_R | TCAAAACTTACCAGCAATCTTCC |  |
| bHLH2_F | ATGGCTGGGCCGCCCAGTAGC | 2103 |
| bHLH2_R | TTAGGGTATTATTTGATGTATTGC |  |
| bHLH3_F | ATGGCTACTACTGGGGTTC | 1932 |
| bHLH3_R | TCAACACTTCCAAATGACTCTG |  |

**Supplementary Table S2｜**Primers used for real-time PCR.

| Name | Sequences(5＇-3＇) | Product size (bp) |
| --- | --- | --- |
| QbHLH1-F | CGAAGCCTGTTTGCTCTG | 168 |
| QbHLH1-R | ATTCATCACCTCCATCCTGA |  |
| QbHLH2-F | CGTTAGTCCCATTCGTCACC | 136 |
| QbHLH2-R | ATCAGCCTCCATCTGCTTGT |  |
| QbHLH3-F | CTGCTGGCTAAGAGTGCTTC | 117 |
| QbHLH3-R | AACCTGCTGAATGAAACTGG |  |
| QACTIN-F | ACCGTATGAGCAAGGAAATCACTG | 160 |
| QACTIN-R | TCGTCGTACTCACCCTTTGAAATC |  |
| QGAPDH-F | GATACAGTTCCCGTGTTGTTGAC | 121 |
| QGAPDH-R | CATAAAGACACATAACACCACACTC |  |

**Supplementary Table S3｜**Primers used for transient expression.

| Name | Sequences(5＇-3＇) | Restriction Site |
| --- | --- | --- |
| Trans-bHLH1_F | TTCTGCCCAAATTCGCGAATGGCTGGTGTTGTTCAAAATCAGG | *Nru* I |
| Trans-bHLH1_R | AGTTAAAGGCCTCGAGTCAAAACTTACCAGCAATCTTCC | *Xho* I |
| Trans-bHLH2_F | TTCTGCCCAAATTCGCGAATGGCTGGGCCGCCCAGTAGC | *Nru* I |
| Trans-bHLH2_R | AGTTAAAGGCCTCGAGTTAGGGTATTATTTGATGTATTGC | *Xho* I |
| Trans-bHLH3_F | TTCTGCCCAAATTCGCGAATGGCTACTACTGGGGTTC | *Nru* I |
| Trans-bHLH3_R | AGTTAAAGGCCTCGAGTCAACACTTCCAAATGACTCTG | *Xho* I |

**Supplementary Table S4｜**Primers used for fusing GFP.

| Name | Sequences(5＇-3＇) | Restriction Site |
| --- | --- | --- |
| MYB1-GFP-F | CAAATTCGCGACCGGTATGTCGCATTTACTTGGTGC | *Age* I |
| MYB1-GFP-R | TGCTAGCCATACCGGTCTTTGCATTGTCTTCTTC | *Age* I |
| bHLH1-GFP-F | CAAATTCGCGACCGGTATGGCTGGTGTTGTTCAAAATC | *Age* I |
| bHLH1-GFP-R | TGCTAGCCATACCGGTAAACTTACCAGCAATCTTCC | *Age* I |
| bHLH2-GFP-F | CAAATTCGCGACCGGTATGGCTGGGCCGCCCAGTAGC | *Age* I |
| bHLH2-GFP-R | TGCTAGCCATACCGGTGGGTATTATTTGATGTATTGC | *Age* I |
| bHLH3-GFP-F | CAAATTCGCGACCGGTATGGCTACTACTGGGGTTC | *Age* I |
| bHLH3-GFP-R | TGCTAGCCATACCGGTACACTTCCAAATGACTCTG | *Age* I |

**Supplementary Table S5｜**Primers used for Yeast Two-Hybrid analysis.

| Name | Sequences(5＇-3＇) | Restriction Site |
| --- | --- | --- |
| AD-bHLH1-F | GGAGGCCAGTGAATTCATGGCTGGTGTTGTTCAAAATC | *Eco*R I |
| AD-bHLH1-R | CGAGCTCGATGGATCCTCAAAACTTACCAGCAATCTTCC | *Bam*H I |
| AD-bHLH2-F | GGAGGCCAGTGAATTCATGGCTGGGCCGCCCAGTA | *Eco*R I |
| AD-bHLH2-R | CGAGCTCGATGGATCCTTAGGGTATTATTTGATGTATTGC | *Bam*H I |
| AD-bHLH3-F | GGAGGCCAGTGAATTCATGGCTACTACTGGGGTTC | *Eco*R I |
| AD-bHLH3-R | CGAGCTCGATGGATCCTCAACACTTCCAAATGACTCTG | *Bam*H I |
| BD-MYB1-F | CATGGAGGCCGAATTCATGTCGCATTTACTTGGTGC | *Eco*R I |
| BD-MYB1-R | GCAGGTCGACGGATCCTTACTTTGCATTGTCTTCTTCTATA | *Bam*H I |
| BD-MYB1A-R | GCAGGTCGACGGATCCACCTGCACACATTGTTCTCA | *Eco*R I |
| BD-MYB1B-R | GCAGGTCGACGGATCCCTCAAATTGACCACTCTCTA | *Eco*R I |
| BD-MYB1C-R | GCAGGTCGACGGATCCATGGTCTCTGCTGTTTTCC | *Eco*R I |
| BD-MYB1D-R | GCAGGTCGACGGATCCAGTACTTGTCATTGTTTTCGAT | *Eco*R I |

**Supplementary Table S6｜**Primers used for BiFC assays.

| Name | Sequences(5＇-3＇) | Restriction Site |
| --- | --- | --- |
| MYB1-NYFP-F | CAAATTCGCGACCGGTATGTCGCATTTACTTGGTGC | *Age* I |
| MYB1-NYFP-R | TGCTCACCATACCGGTCTTTGCATTGTCTTCTTC | *Age* I |
| bHLH1-CYFP-F | CAAATTCGCGACCGGTATGGCTGGTGTTGTTCAAAATC | *Age* I |
| bHLH1-CYFP-R | GCACGCTGCCACCGGTAAACTTACCAGCAATCTTCC | *Age* I |
| bHLH2-CYFP-F | CAAATTCGCGACCGGTATGGCTGGGCCGCCCAGTAGC | *Age* I |
| bHLH2-CYFP-R | GCACGCTGCCACCGGTGGGTATTATTTGATGTATTGC | *Age* I |
| bHLH3-CYFP-F | CAAATTCGCGACCGGTATGGCTACTACTGGGGTTC | *Age* I |
| bHLH3-CYFP-R | GCACGCTGCCACCGGTACACTTCCAAATGACTCTG | *Age* I |

**Supplementary Table S7｜**Primers used for dual luciferase transient tobacco assay.

| Name | Sequences(5＇-3＇) | Restriction Site |
| --- | --- | --- |
| CHSp-F | TATAGGGCGAATTGGGTACCCTGGATTAGAAATGGGAAGG | *Knp* I |
| CHSp-R | TAGAACTAGTGGATCCTTTTCCCTAGCTTCAAGAGAAAAACTG | *Bam*H I |
| CHIp-F | TATAGGGCGAATTGGGTACCGTCTCGTCGAATGATTAGAATTG | *Knp* I |
| CHIp-R | TAGAACTAGTGGATCCTATTCACAGGCTGTTGCGTTCAG | *Bam*H I |
| F3Hp-F | TATAGGGCGAATTGGGTACCCCCACACGTCTTAATGACCAAC | *Knp* I |
| F3Hp-R | TAGAACTAGTGGATCCTCACACAAGTTGTTTTTTTGGTGTG | *Bam*H I |
| F3'Hp-F | TATAGGGCGAATTGGGTACCACGGCTTTGTAATCATGCAG | *Knp* I |
| F3'Hp-R | TAGAACTAGTGGATCCAGTTACAAGGATTGATGGAGGAG | *Bam*H I |
| DFRp-F | TATAGGGCGAATTGGGTACCCTACTCTGGTTTAATTGGGAGC | *Knp* I |
| DFR p-R | TAGAACTAGTGGATCCACACAGTCTCAGATTGTGATCCCAT | *Bam*H I |
| ANSp-F | TATAGGGCGAATTGGGTACCCGTAGGAAAATCGTGAGGTTG | *Knp* I |
| ANSp-R | TAGAACTAGTGGATCCCTCTGTCAACGTAATTTTCTG | *Bam*H I |
| UFGTp-F | TATAGGGCGAATTGGGTACCTATACGAGCAAGAAAAGACTGAAGC | *Knp* I |
| UFGTp-R | TAGAACTAGTGGATCCTATATACATATGTATAGACATGTATATG | *Bam*H I |


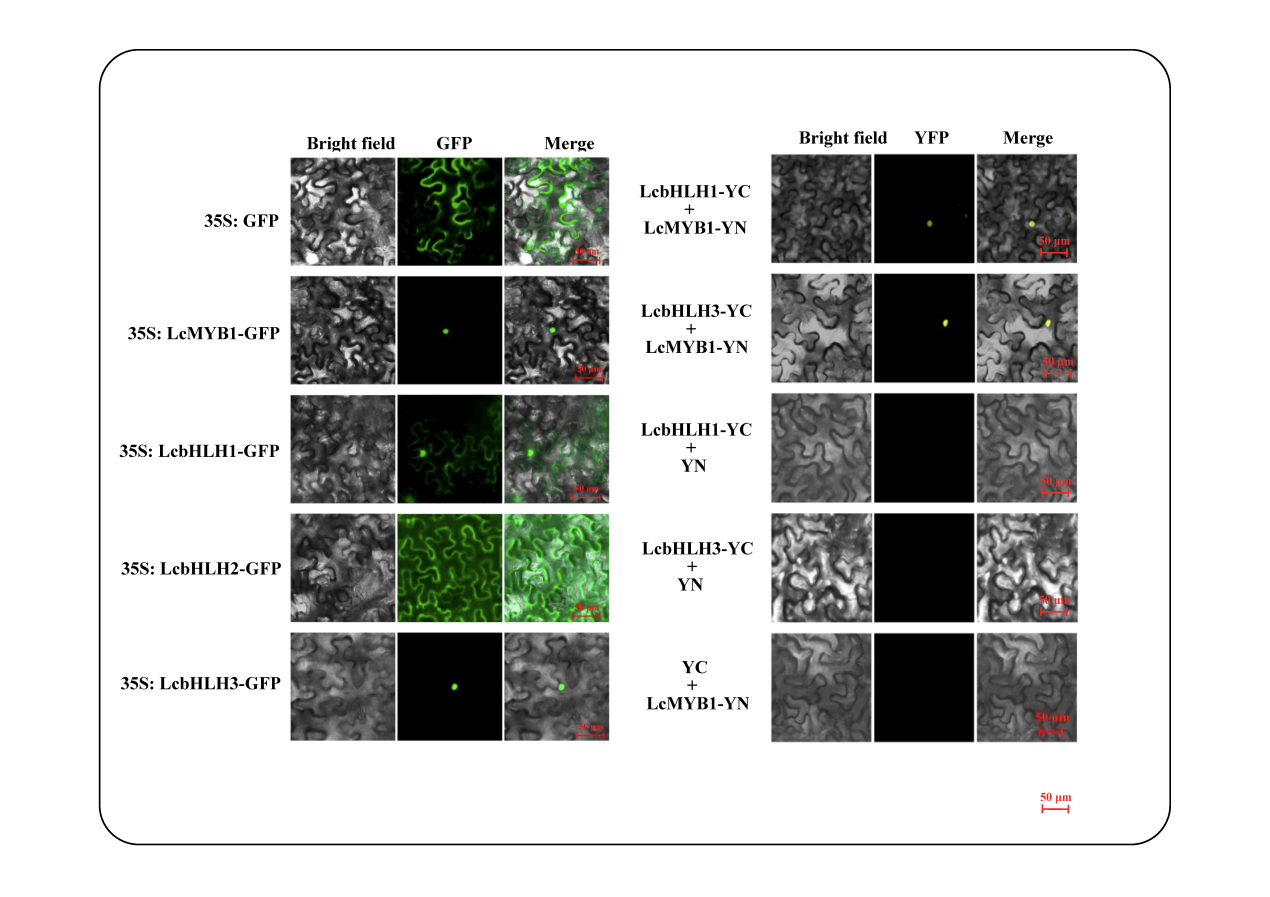


**Supplementary Figure S1｜**Epidermal cells of *Nicotiana benthamiana* leaves were transiently transformed with LcMYB1–GFP and LcbHLHs–GFP constructs in *Agrobacterium tumefaciens* strain GV3101. GFP fluorescence was observed with a fluorescence microscope. Images were taken in a dark field for green fluorescence, while the outline of the cell and the merged were photographed in a bright field. Bars, 50 μm.


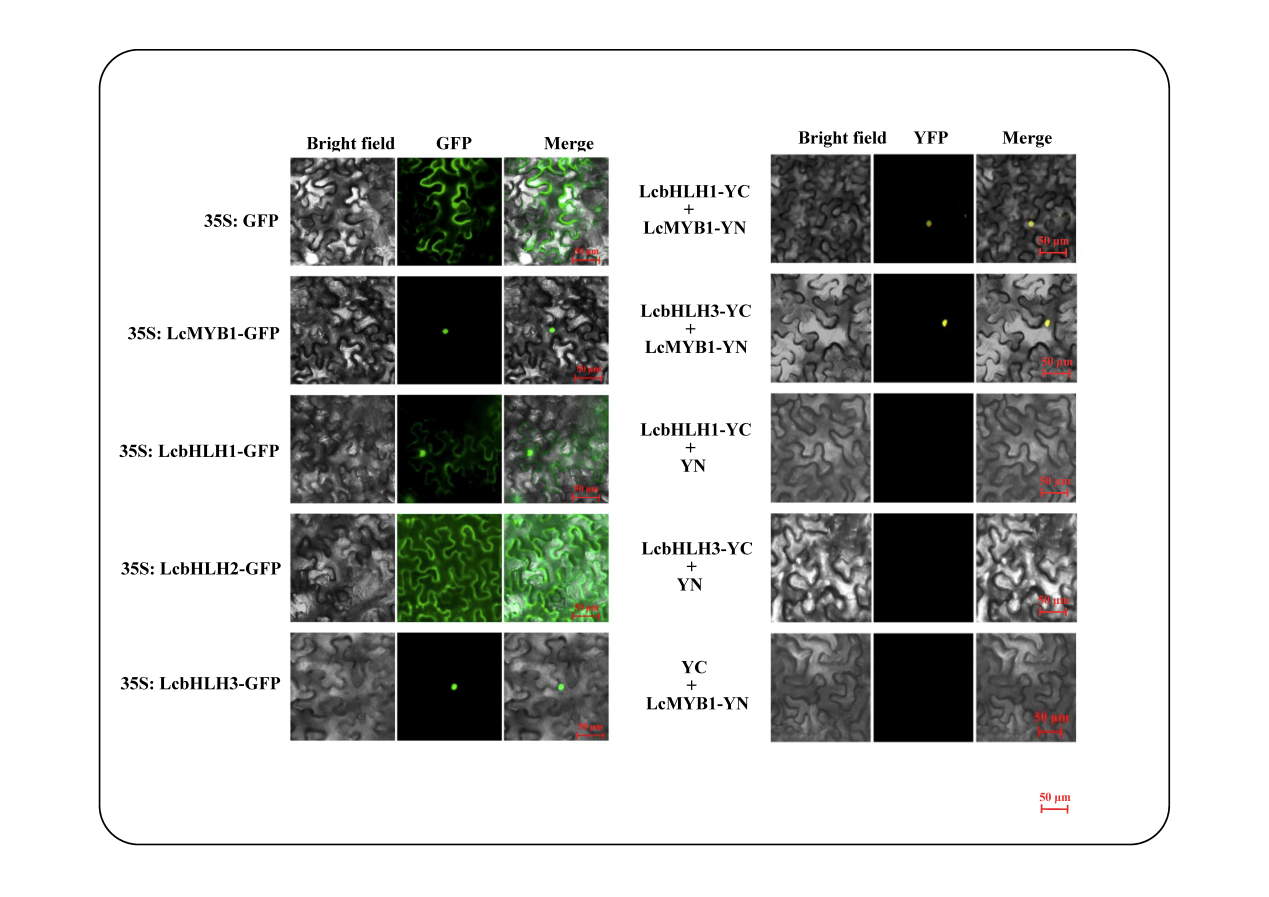


**Supplementary Figure S2｜**Bimolecular fluorescence complementation (BiFC) visualization of the LcbHLH1 and LcbHLH3 interaction in transiently co-expressed in *Nicotiana benthamiana* leaf epidermic cells. YFP indicates fluorescence of YFP; Merge is digital merge of bright field and fluorescent images. Bars, 50 μm.


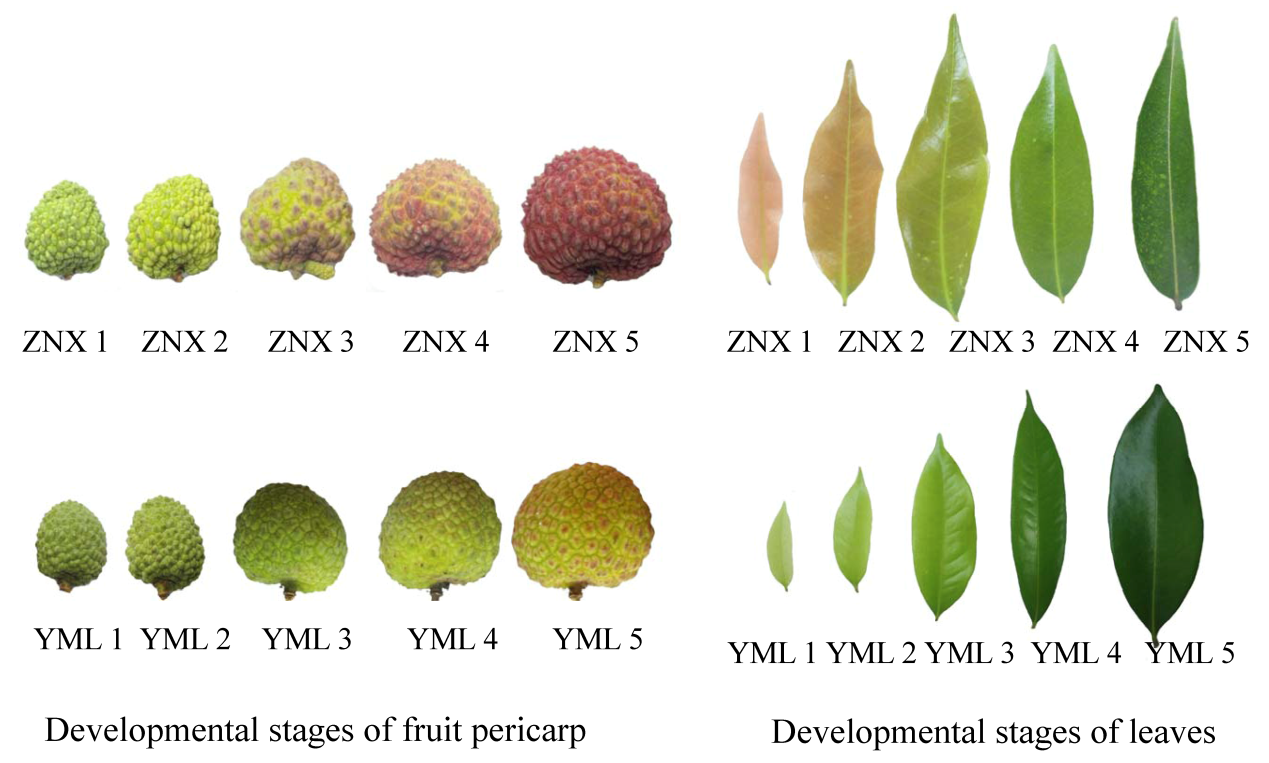


**Supplementary Figure S3｜**Development stages of ZNX (Ziniangxi) and YML (Yamulong) fruit and leaf.


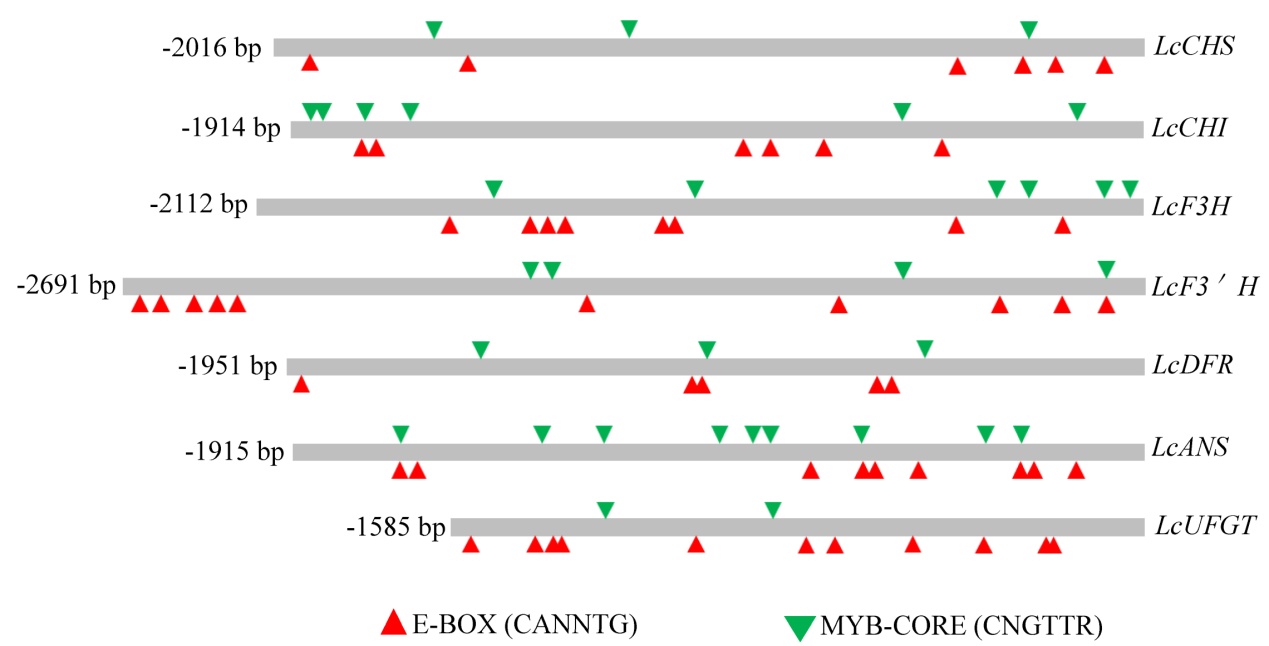


**Supplementary Figure S4｜**E-BOX and MYB-CORE conserve sequences in promoter of litchi anthocyanin biosynthesis genes.
